# Community pharmacy services and patient quality of life in Lebanon's socioeconomic crisis: Findings from the IMPHACT-LB study

**DOI:** 10.1016/j.rcsop.2025.100659

**Published:** 2025-09-22

**Authors:** Aline Hajj, Marwan Akel, Rony M. Zeenny, Hala Sacre, Chadia Haddad, Jihan Safwan, Fouad Sakr, Pascale Salameh

**Affiliations:** a INSPECT-LB (Institut National de Santé Publique, d'Épidémiologie Clinique et de Toxicologie-Liban), Beirut, Lebanon; b Faculté de Pharmacie, Université Laval, Québec, Canada; c Oncology Division, CHU de Québec Université Laval Research Center, Québec, Canada; d Department of Pharmacy, American University of Beirut Medical Center, Beirut, Lebanon; e Drug Information Center, Order of Pharmacists of Lebanon, Beirut, Lebanon; f Faculty of Public Health, Lebanese University, Fanar, Lebanon; g Research Department, Psychiatric Hospital of the Cross, Jal Eddib, Lebanon; h Inserm U1094, IRD UMR270, EpiMaCT Epidemiology of Chronic Diseases in Tropical Zone, University of Limoges, Limoges, France; i School of Pharmacy, Lebanese International University, Beirut, Lebanon; j School of Medicine, Lebanese American University, Byblos, Lebanon; k Faculty of Pharmacy, Lebanese University, Hadat, Lebanon; l Department of Primary Care and Population Health, University of Nicosia Medical School, 2417 Nicosia, Cyprus

**Keywords:** Community pharmacy, Pharmacy service, Patient, Quality of life, Well-being

## Abstract

**Background:**

In crisis-affected settings like Lebanon, community pharmacists face mounting challenges in securing quality medications and supporting patient care. This study aims to explore the association between pharmacy services, pharmacist-patient relationships, and patient quality of life during Lebanon's ongoing socioeconomic collapse.

**Methods:**

A cross-sectional study was conducted in April 2023, using an online convenience sampling. Validated tools, including the 5-Level EuroQol (EQ-5D-5L) and EQ visual analog scale (EQ-VAS), were used to assess quality of life among a sample of 865 Lebanese adults recruited via social media platforms. Due to the absence of a national census, random sampling was not feasible, limiting generalizability.

**Results:**

Higher EQ-VAS scores were significantly associated with better financial well-being (Beta = 0.18, *p* < 0.001), high monthly income (Beta = 7.04, p < 0.001), regular pharmacy visits (Beta = 3.06, *p* = 0.035), and perceiving pharmacists as medication counselors (Beta = 5.43; *p* = 0.003). Lower scores were associated with a higher number of chronic diseases (Beta = −2.66; p < 0.001), obtaining chronic medications from the pharmacy (Beta = −6.20), frequent pharmacy visits for medical care or counseling (Beta = −7.00; p = 0.003), spending more than 10 min with the pharmacist for counseling about a medication and/or medical condition (Beta = −6.31).

**Conclusion:**

This study uniquely quantifies the association between pharmacist-patient interactions and quality of life in a context of systemic disruption. While previous literature has acknowledged pharmacists' roles, our findings demonstrate that their perceived counseling function and continuity of care are independently associated with improved well-being, even after adjusting for socioeconomic and health-related factors.

## List of Abbreviations

**Table:** 

COPD	Chronic Obstructive Pulmonary Disease
EQ-5D-5L	5-Level EuroQol
FIP	International Pharmaceutical Federation
IFDFW	InCharge Financial Distress/Financial
IMPHACT-LB	Impact of the Modern PHArmacy Concept on Therapy of patients in Lebanon
LBP	Lebanese Pounds
MTM	medication therapy management
SCF-CP	specialized competencies framework for community pharmacists
SPSS	Statistical Package for Social Sciences
VIF	Variance Inflation Factor

## 1 Introduction

Community pharmacists have traditionally been essential providers in healthcare systems, delivering accessible services such as medication dispensing, counseling, and chronic disease management.^1^, 2., 3, 4, 5

Over the past decades, their role has evolved toward patient-centered care,^1^^,^^6^ which emphasizes individualized, holistic services that empower patients to actively manage their health and treatment decisions.^7^ This evolution has been linked to improved therapeutic outcomes, enhanced patient satisfaction, and better medication adherence.^1^

In Lebanon, community pharmacies have similarly expanded their scope from dispensing medications to becoming centers of patient care.^8^^,^^9^ However, the country is facing an unprecedented combination of economic collapse, political instability, and healthcare system deterioration.^10^^,^^11^ These crises have disrupted medication supply chains, increased drug costs, reduced access to healthcare services, and eroded public trust in the system.^12^ Consequently, patients often delay or forego care, seek unregulated alternatives, or rely heavily on pharmacists for guidance. These shifts in health-seeking behavior make the pharmacist's role post-crisis uniquely critical, yet prior studies have not systematically assessed this impact.^13^

International evidence also illustrates how pharmacists adapt in crisis-affected settings. For example, during Greece's financial crisis, patients increasingly relied on community pharmacies for primary care due to reduced access to hospitals and physicians.14., 15, 16 In Venezuela, widespread medication shortages forced pharmacists to actively manage therapeutic alternatives and provide expanded patient education. These cases highlight how pharmacy services can maintain continuity of care and safeguard health outcomes in disrupted systems, providing a relevant comparative context for Lebanon.

Previous Lebanese studies, including a pilot in Beirut (2018–2019), showed that patient satisfaction was associated with improved quality of life. However, these studies were conducted before the current crises and were geographically limited, leaving a gap in understanding how pharmacy services function under severe socioeconomic stress.^17^ Moreover, recent analyses indicate that quality of life is primarily associated with sociodemographic factors in the current Lebanese context, with the role of pharmacists remaining unclear and complex.^18^

A range of theoretical frameworks has been employed to conceptualize pharmacy services and healthcare utilization. Donabedian's Quality of Care model,^19^ for example, frames service delivery through the interrelated dimensions of structure, process, and outcomes, while Andersen's Behavioral Model of Health Services Use^20^^,^^21^ underscores the influence of individual, provider, and contextual factors on patterns of care-seeking. Patient-centered pharmacy service models^22^ extend this perspective by emphasizing holistic care and patient empowerment. The current research was inspired by the Pharmaceutical Care Practice model, which emphasizes the pharmacist's role in optimizing medication therapy outcomes through direct patient care.^23^ The pillars of the Pharmaceutical Care practice framework can be summarized as: a professional patient-pharmacist relationship, systematic data collection, therapeutic plan development, tailored intervention implementation, and regular follow-up to optimize patient outcomes.^23^

This framework guided our approach by helping us to better understand the links between pharmacy services and measurable patient outcomes, such as quality of life in Lebanon. The current study examined the association between community pharmacy services, pharmacist-patients interactions, and patient quality of life, explicitly considering the challenging socioeconomic context in Lebanon. Hence, this research would provide novel insights into the evolving role of pharmacists in maintaining health outcomes and patient well-being amidst systemic disruptions.

## 2 Methods

### 2.1 Study design

This cross-sectional study is conducted as part of a larger project, the Impact of the Modern PHArmacy Concept on Therapy of patients in Lebanon: The IMPHACT-LB study.^24^ It involved 865 Lebanese adults recruited between April 11 and April 27, 2023. The survey instrument was created on Google Forms and distributed through various social platforms, such as WhatsApp, Facebook, and Instagram, to reach potential participants from all Lebanese Governorates, i.e., Beirut, Mount Lebanon, North, South, and Beqaa. We note that in Lebanon, there is no sampling framework for adults due to the absence of a census in the country since 1932^25^; in this situation, and given the impossibility of selecting a random sample, the use of a convenience sample allowed for a fast reach of a diverse sample of the target population. The short data collection period allowed for capturing a static snapshot of the general population's opinion.

The study targeted Lebanese individuals aged 18 and above, whose participation was entirely voluntary. They had the freedom to withdraw from the study at any point. Participants received no incentives and provided written informed consent before engaging in the study.

### 2.2 Sample size calculation

The minimum sample size was calculated using the G-Power software, version 3.0.10. The calculated effect size was 0.0526, expecting a squared multiple correlation of 0.05 (R^2^ deviation from 0) related to the Omnibus test of multiple regression. The minimum sample required was 454 participants, considering a 5 % alpha error, 80 % power, and the inclusion of 25 predictors in the model. A minimum of 600 participants was targeted to account for potential missing values.

### 2.3 Survey tool

The survey questionnaire was available in Arabic, the native language of Lebanon. It comprised three sections.

The first section focused on the participants' sociodemographic characteristics, such as age, education level, occupation, and income. It also inquired about their medical condition, health coverage, access to healthcare services, and the primary reasons for visiting a community pharmacy.

The second section explored participants' experiences with community pharmacy visits. It included questions about the services provided by community pharmacists, overall perceptions and experiences with these services, frequency of pharmacy visits, and loyalty to a specific pharmacy. These questions drew inspiration from previous research (30,31).

The third part consisted of several validated measures:

#### 2.3.1 The InCharge Financial Distress/Financial (IFDFW) scale

In this study, the Arabic version of the IFDFW scale previously validated in Lebanon was used to assess the effect of the current economic crisis.^26^ This 8-item tool measures financial distress and well-being on a linear scale ranging from 1 to 10. Higher scores indicate better financial well-being.^27^

#### 2.3.2 The 5-Level EuroQol (EQ-5D-5L)

The EQ-5D-5L developed by the EuroQol Group assessed patients' overall health and well-being.^28^ This standardized measure comprises the EQ-5D descriptive system and the EQ visual analog scale (EQ VAS). EQ-5D covers five dimensions, i.e., mobility, self-care, usual activities, pain/discomfort, and anxiety/depression, each measured on five levels: (1) no problems, (2) slight problems, (3) moderate problems, (4) severe problems, and (5) extreme problems. Respondents indicate their health state by choosing the most appropriate statement in the five dimensions. In this study, its Cronbach alpha reliability measure was 0.753. In addition, EQ VAS measures health status ranging from 0 (worst imaginable health) to 100 (best imaginable health).

### 2.4 Statistical analysis

The collected data were analyzed using Statistical Package for Social Sciences (SPSS) software, version 28.0. For descriptive analysis, frequency and percentage were used for categorical variables, and mean and standard deviation for quantitative variables. The Cronbach alpha was first calculated to confirm the scales' reliability in the current sample. No missing values were found in the database, as the online platform required answering all the questions.

Since the dependent variable was continuous, the distribution was considered normal using visual inspection of the histogram, while the skewness and kurtosis were lower than 1. These conditions are compatible with normality with a sample size higher than 300 (which is the case of this study sample).

For the bivariate analysis, the Student's *t*-test was used to compare the means between 2 groups and ANOVA to compare between three groups or more after checking for homogeneity of variances using Levene's test. If the variances are not homogenous, the corrected t-test and the Kruskal-Wallis test are used, respectively. After ANOVA and Kruskal-Wallis significant testing, post hoc analyses were conducted using Bonferroni adjustment. A Pearson correlation coefficient was used between continuous variables. In all cases, a *p*-value lower than 0.05 was considered significant.

As for the multivariable analysis, multiple linear regressions were conducted to assess the correlates of the dependent variable in the sample after checking the residues' normality, the linearity of the relationship, the absence of multicollinearity, and the homoscedasticity assumptions. The “Enter” method was used in the first model, considering the EQ Visual Analogue Scale as the dependent variable and the sociodemographic variables as the independent variables. In the second model, the pharmacist-related variables were added to the model, and the analysis was run using the “stepwise method.” In both models, variables related to disease status were included to remove the confounding effect on quality of life. The beta coefficient, 95 % Confidence Interval, and the *p*-value were reported. Independent variables introduced in the models were those having a p-value lower than 0.1 in the bivariate analysis, taking into account the maximum number allowed of variables to be included, given the sample size: sociodemographic, and other independent variables were added as appropriate. This method is compatible with a strict, purposeful selection of covariates to be included in the multivariable model, leading to a parsimonious model, as described by Bursac and collaborators.^29^

## 3 Results

### 3.1 Sample description

This study included 865 participants, whose sociodemographic details are shown in Table 1. More than half of the participants (68.8 %) were females, 60.1 % were single, 78.3 % had a university degree, 51.1 % were unemployed, 52.5 % earned more than 135 US$ per month, and 45.9 % lived in the Beirut and Mount Lebanon areas. Only 19.5 % reported having chronic medical conditions, with a mean of 1.42 ± 2.16 chronic diseases. Among them, 25.1 % had allergies, 14.1 % suffered from depression, anxiety, or other psychiatric disorders, and 14.1 % reported chronic gastrointestinal illnesses. Moreover, 77.6 % reported having easy access to healthcare, while 29.2 % were covered by private insurance. The mean age was 32.52 ± 14.56, and the mean number of medications taken routinely per day was 0.87 ± 1.78.

**Table 1:** Sociodemographic and medical characteristics of participants (*N* = 865).

Variable	N (%)
**Gender**	
Male	270 (31.2 %)
Female	595 (68.8 %)
**Area of residence**	
Beirut	215 (24.9 %)
Mount Lebanon	182 (21.0 %)
North	151 (17.5 %)
Beqaa	17 (2.0 %)
South	300 (34.7 %)
**Marital status**	
Single/widowed/divorced	520 (60.1 %)
Married	345 (39.9 %)
Monthly income**	
Less than 4,000,000 LBP (Equivalent to 45 US$)	44 (5.1 %)
Between 4,000,000–8,000,000 LBP (45–90 US$)	176 (20.3 %)
Between 8,000,000–12,000,000 LBP (90–135 US$)	191 (22.1 %)
More than 12,000,000 LBP (>135 US$)	454 (52.5 %)
**Education level**	
Illiterate	16 (1.8 %)
School level	172 (19.9 %)
University level	677 (78.3 %)
**Work status**	
Unemployed	442 (51.1 %)
Employed	407 (47.1 %)
Retired	16 (1.8 %)
**Health status perception**	
No sickness	686 (79.3 %)
Chronic medical condition	169 (19.5 %)
Severe sickness	10 (1.2 %)
Presence of a medical illness*	
Hypertension	97 (11.2 %)
Diabetes mellitus	58 (6.7 %)
Dyslipidemia	85 (9.8 %)
Cardiovascular disease	51 (5.9 %)
Chronic kidney disease	21 (2.4 %)
Asthma	51 (5.9 %)
COPD	10 (1.2 %)
Other chronic pulmonary diseases	13 (1.5 %)
Allergic conditions	217 (25.1 %)
History of stroke or cerebrovascular diseases	19 (2.2 %)
Seizures or other neurological conditions	37 (4.3 %)
Depression, anxiety, or other psychiatric conditions	122 (14.1 %)
Chronic gastrointestinal conditions	122 (14.1 %)
Current or previous cancer	17 (2.0 %)
Rheumatic disease	43 (5.0 %)
Other chronic diseases	51 (5.9 %)
**Easy access to healthcare**	
Yes	671 (77.6 %)
No	194 (22.4 %)
	**Mean ± SD**
**Age**	32.52 ± 14.56
**Financial well-being scale**	39.88 ± 18.11
**Number of chronic diseases**	1.42 ± 2.16
**Number of medications taken routinely per day**	0.87 ± 1.78

*Multiple answers are possible; ** 1 US$ is equivalent to 90,000LBP

LBP: Lebanese Pounds; COPD: Chronic Obstructive Pulmonary Disease

### 3.2 Description of community pharmacy visits and the pharmacist-patient relationship

Of all participants, nearly half visited the community pharmacy frequently, went to the pharmacy to buy non-prescription drugs, and were loyal to the same pharmacy; a few (11 %) visited it weekly for medical attention or counseling (Table 2). When participants were asked about the frequency of their visits for specific reasons, almost half of them said they did so to buy non-prescription drugs, one fourth to seek initial medical assessment and/or care, and one fourth to get general or specific medical advice. Also, one-third preferred to talk to the pharmacist about their medical conditions. The main reasons for visiting a community pharmacy were mainly to inquire about medications (68 %), inquire about a sickness (36.0 %), and receive treatment from the pharmacist (46 %). One third of participants reported that the pharmacy had a counseling area, half said the counseling sessions lasted 5 to 10 min, and half admitted they occasionally received counseling from the pharmacist. Most participants perceived the pharmacist as a medicine expert (94 %), a health counselor (81 %), a health advocate (66 %), and a patient-centered practitioner (73 %). Lastly, 72 % received recommendations for a healthy lifestyle from the pharmacist, and 80 % reported that the pharmacist offered extra healthcare services.

**Table 2:** Community pharmacy visits and pharmacist-patient relationship (*N* = 865).

Variable	N (%)
**Are you familiar with the current community pharmacy?**	
Recent visitor (< 1 year)	192 (22.2 %)
Regular visitor (1–5 years)	333 (38.5 %)
Chronic visitor (> 5 years)	340 (39.3 %)
**Do you visit the same pharmacy each time?**	
Yes, the same pharmacy each time	440 (50.9 %)
No, different pharmacies depending on the situation/ circumstances	425 (49.1 %)
**How frequently do you visit a community pharmacy for medical care or counseling?**	
Daily	16 (1.8 %)
Weekly	92 (10.6 %)
Monthly	349 (40.3 %)
Rarely	408 (47.2 %)
**How frequently do you visit your community pharmacy to obtain non-prescription medications?**	
Always	71 (8.2 %)
Most of the time	283 (32.7 %)
Sometimes	169 (19.5 %)
Rarely	302 (34.9 %)
Never	40 (4.6 %)
**How frequently do you visit your community pharmacy to seek initial medical assessment and/or care?**	
Always	34 (3.9 %)
Most of the time	168 (19.4 %)
Sometimes	259 (29.9 %)
Rarely	284 (32.8 %)
Never	120 (13.9 %)
**How frequently do you visit your community pharmacy for general or specific medical advice?**	
Always	42 (4.9 %)
Most of the time	173 (20.0 %)
Sometimes	258 (29.8 %)
Rarely	279 (32.3 %)
Never	113 (13.1 %)
**How frequently do you prefer discussing your medical conditions with your pharmacist before referring to your primary care provider or doctor?**	
Always	86 (9.9 %)
Most of the time	229 (26.5 %)
Sometimes	204 (23.6 %)
Rarely	241 (27.9 %)
Never	105 (12.1 %)
**Does the pharmacy you visit include a counseling area?**	
I do not know	269 (31.1 %)
No	309 (35.7 %)
Yes	287 (33.2 %)
**Do you mainly come to a community pharmacy to ask about medications?**	
No	275 (31.8 %)
Yes	590 (68.2 %)
**Do you mainly come to a community pharmacy to ask about a disease?**	
No	554 (64.0 %)
Yes	311 (36.0 %)
**Do you mainly come to a community pharmacy to receive treatment from the pharmacist?**	
No	469 (54.2 %)
Yes	396 (45.8 %)
**Do you receive regular counseling from your pharmacist?**	
No, not at all	252 (29.1 %)
Yes, regularly	174 (20.1 %)
Yes, from time to time	439 (50.8 %)
**How long does the pharmacist spend counseling you about a medication and/or medical condition?**	
Less than 5 min	372 (45.8 %)
5 to 10 min	387 (47.6 %)
More than 10 min	54 (6.6 %)
**Do you perceive your pharmacist as a medication expert?**	
No	50 (5.8 %)
Yes	815 (94.2 %)
**Do you perceive your pharmacist as a health counselor?**	
No	164 (19.0 %)
Yes	701 (81.0 %)
**Do you perceive your pharmacist as a health promoter?**	
No	292 (33.8 %)
Yes	573 (66.2 %)
**Do you perceive your pharmacist as a patient-centered practitioner?**	
No	231 (26.7 %)
Yes	634 (73.3 %)
**Does your pharmacist advise you about healthy lifestyle modifications, including smoking cessation, weight loss, or physical activity?**	
No	244 (28.2 %)
Yes	621 (71.8 %)
**Does your pharmacist provide additional healthcare services like measuring blood pressure and/or glucose?**	
No	175 (20.2 %)
Yes	690 (79.8 %)

### 3.3 Description of the EQ-5D-5L health questionnaire

A minority of participants have problems with mobility (24.4 %), self-care (7.2 %), and usual activity (21.6 %). However, about half of the sample reported having anxiety/depression (52 %) and pain discomfort (47 %). The mean EQ-5D-5L was 7.26 ± 2.65, while the EQ-VAS was 73.60 ± 23.07 (Table 3). The histogram of the EQ-VAS logarithm showed a normal distribution, while that of the EQ-5D-5L did not (results not shown); thus, the EQ-VAS was used for the rest of the analysis.

**Table 3:** Description of the EQ-5D-5L health questionnaire.

		Frequency (%)
Mobility	Absence of a problem	654 (75.6 %)
	Presence of a problem	211 (24.4 %)
Self-care	Absence of a problem	803 (92.8 %)
	Presence of a problem	62 (7.2 %)
Usual activities	Absence of a problem	678 (78.4 %)
	Presence of a problem	187 (21.6 %)
Pain discomfort	Absence of a problem	459 (53.1 %)
	Presence of a problem	406 (46.9 %)
Anxiety/depression	Absence of a problem	416 (48.1 %)
	Presence of a problem	449 (51.9 %)
**EQ-5D-5L total**		7.26 ± 2.65 [Min 5; Max 19]
**EQ visual analogue scale (EQ-VAS)**		73.60 ± 23.07 [Min 0; Max 100]

EQ-5D-5L: 5- Level EuroQol.

### 3.4 Bivariate analysis

Table 4 shows the bivariate analysis using the EQ-VAS scale as the dependent variable. A significantly higher mean EQ VAS score was found among participants who had a high income, a university education level, no medical conditions, and easy access to healthcare and those who rarely visited the community pharmacy, sometimes visited the community pharmacy to seek initial medical assessment and general or specific medical advice, received less than 5 min of counseling from the pharmacist, perceived the pharmacist as a health counselor, and those who had other reasons for visiting a community pharmacy. A significantly higher age and number of chronic diseases and medications were associated with lower EQ-VAS scores. The financial well-being scale was significantly associated with a higher EQ-VAS score.

**Table 4:** Bivariate analysis using the EQ Visual Analogue Scale as the dependent variable.

Variable	EQ VAS Mean ± SD	p-value
**Gender**		
Male	75.62 ± 22.21	0.084
Female	72.68 ± 23.42	
**Marital status**		
Single/widowed/divorced	74.83 ± 23.64	0.056
Married	71.75 ± 22.09	
**Monthly income**		
Low (Less than 4,000,000 LBP) (Equivalent to 45 $US)	59.72 ± 28.94	<0.001
Intermediate (Between 4,000,000–12,000,000 LBP) (45–135 $US)	69.20 ± 24.60	
High (More than 12,000,000 LBP) (>135 $US)	78.51 ± 19.72	
**Education level**		
School level	69.11 ± 24.88	0.003
University level	74.83 ± 22.42	
**Work status**		
Unemployed	73.04 ± 23.42	0.449
Employed	74.24 ± 22.69	
**Health status**		
No sickness	76.43 ± 22.73	<0.001
Medical conditions	62.77 ± 21.16	
**Easy access to healthcare**		
Yes	75.69 ± 21.75	<0.001
No	66.40 ± 25.95	
**Familiar with the current community pharmacy**		
Recent visitor (< 1 year)	71.73 ± 26.12	0.097
Regular visitor (1–5 years)	75.71 ± 22.28	
Chronic visitor (> 5 years)	72.58 ± 21.92	
**How frequently do you visit a community pharmacy for medical care or counseling**		
Daily	60.56 ± 35.74	<0.001
Weekly (less than daily and up to once a week)	63.09 ± 28.82	
Monthly (less than weekly and up to once a month)	71.15 ± 22.27	
Rarely (less than once a month)	78.57 ± 20.30	
**How frequently do you visit your community pharmacy to obtain non-prescription medications?**		
Always	71.57 ± 26.60	0.060
Most of the time	72.83 ± 21.22	
Sometimes	76.00 ± 23.60	
Rarely	72.35 ± 23.77	
Never	82.15 ± 19.66	
**How frequently do you visit your community pharmacy to seek initial medical assessment and/or care?**		
Always	70.41 ± 25.70	0.040
Most of the time	70.19 ± 23.39	
Sometimes	76.83 ± 22.27	
Rarely	72.63 ± 23.04	
Never	74.55 ± 23.07	
**How frequently do you visit your community pharmacy for general or specific medical advice?**		
Always	69.47 ± 28.14	<0.001
Most of the time	68.84 ± 25.06	
Sometimes	78.18 ± 19.69	
Rarely	71.89 ± 24.05	
Never	76.18 ± 20.61	
**Do you mainly come to a community pharmacy to ask about medications?**		
No	76.11 ± 23.69	0.030
Yes	72.43 ± 22.70	
**How long does the pharmacist spend counseling you about a medication and/or medical condition?**		
Less than 5 min	75.17 ± 20.67	0.025
5 to 10 min	72.57 ± 24.99	
More than 10 min	66.46 ± 26.93	
**Do you perceive your pharmacist as a medication expert?**		
No	65.14 ± 35.77	0.092
Yes	74.11 ± 22.03	
**Do you perceive your pharmacist as a health counselor?**		
No	69.15 ± 29.19	0.025
Yes	74.64 ± 21.30	
**Do you perceive your pharmacist as a patient-centered practitioner?**		
No	70.93 ± 26.32	0.062
Yes	74.57 ± 21.72	
**What is the primary reason for visiting a community pharmacy?**		
Obtain chronic medications (hypertension, diabetes mellitus, cardiovascular, etc.)	59.72 ± 27.07	<0.001
Obtain non-prescription medications (analgesics, anti-inflammatory drugs, supplements, etc.)	76.22 ± 22.43	
Obtain both chronic and non-prescription medications	69.68 ± 18.69	
Other reason	76.74 ± 23.84	
	**Correlation**	
**Age**	−0.139	<0.001
**Financial well-being scale**	0.249	<0.001
**Number of chronic diseases**	−0.343	<0.001
**Number of medications taken routinely per day**	−0.260	<0.001

### 3.5 Multivariable analysis

A first linear regression model with the EQ-VAS scale as the dependent variable and the sociodemographic variables as independent variables showed that a high monthly income (Beta = 10.92), easy access to healthcare (Beta = 4.16), and higher financial well-being (Beta = 0.17) were significantly associated with a higher EQ-VAS score. However, having many chronic diseases (Beta = −2.81) was significantly associated with a lower EQ-VAS score. From the effect size perspective, the two most important correlates were the number of chronic diseases and the monthly income, while the easy access to healthcare had a low effect on quality of life (Table 5, Model 1).

**Table 5:** Multivariable analysis.

Model 1: Linear regression analysis using the EQ-VAS as the dependent variable and sociodemographic characteristics as the independent variable						
	UB	SB.	p-value	95 % Confidence Interval		VIF
				Lower bound	Upper bound	
Gender (female vs. male*)	−1.509	−0.030	0.337	−4.592	1.575	1.051
Marital status (married vs. single*)	0.532	0.011	0.777	−3.146	4.209	1.662
Monthly income (intermediate vs. low)	4.744	0.102	0.165	−1.960	11.448	5.638
**Monthly income (high vs. low)**	**10.924**	**0.237**	**0.002**	**4.130**	**17.718**	5.909
Education level (university vs. school*)	2.515	0.045	0.180	−1.166	6.196	1.170
Health status (presence vs. absence of a medical condition *)	−2.543	−0.045	0.279	−7.145	2.060	1.784
Easy access to healthcare (yes vs. no*)	**4.166**	**0.075**	**0.020**	**0.665**	**7.666**	1.094
Age	−0.038	−0.024	0.595	−0.178	0.102	2.138
**Financial well-being scale**	**0.175**	**0.137**	**<0.001**	**0.091**	**0.259**	1.195
**Number of chronic diseases**	**−2.816**	**−0.263**	**<0.001**	**−3.612**	**−2.021**	1.509


Model 2: Linear regression analysis using the EQ-VAS as the dependent variable and sociodemographic and pharmacist-related characteristics as the independent variables						
	UB	SB.	p-value	95 % Confidence Interval		VIF
				Lower bound	Upper bound	
Number of chronic diseases	−2.661	−0.249	<0.001	−3.354	−1.968	1.193
Financial well-being scale	0.189	0.148	<0.001	0.105	0.272	1.228
High monthly income	7.045	0.153	<0.001	4.129	9.961	1.135
Reason for visiting pharmacy; obtain chronic medications (hypertension, diabetes mellitus, cardiovascular, etc.)	−6.205	−0.079	0.016	−11.236	−1.174	1.156
Do you perceive the pharmacist as a medication counselor (yes vs. no*)	5.431	0.092	0.003	1.902	8.960	1.019
How frequently do you visit a community pharmacy for medical care or counseling (weekly vs. rarely*)	−7.001	−0.093	0.003	−11.584	−2.417	1.054
How frequently do you visit your community pharmacy for general or specific medical advice (sometimes vs. never*)	3.454	0.069	0.027	0.401	6.506	1.045
Time the pharmacist spends on counseling you about a medication and/or medical condition (more than 10 min vs. less than 5 min*)	−6.312	−0.066	0.030	−12.015	−0.609	1.025
How frequently do you visit your community pharmacy to obtain non-prescription medications (rarely vs. never*)	−3.832	−0.079	0.011	−6.767	−0.897	1.047
How familiar are you with the current community pharmacy: regular visitor (1–5 years) vs. recent visitor (< 1 year)	3.069	0.065	0.035	0.214	5.924	1.033
Easy access to healthcare (yes vs. no*)	3.625	0.066	0.039	0.185	7.064	1.103

Variables entered in the model: gender, marital status, monthly income, education level, health status, easy access to healthcare, age, financial well-being scale, and Number of chronic diseases.

Variables entered in the model: gender, marital status, monthly income, education level, health status, easy access to healthcare, age, financial well-being scale, Number of chronic diseases, Familiar with the current community pharmacy, frequent visits community pharmacy for medical care or counseling, visit community pharmacy to obtain non-prescription medications, visit community pharmacy to seek initial medical assessment and/or care, visit your community pharmacy for general or specific medical advice, come to a community pharmacy to ask about medications, time spend counseling about a medication and/or medical condition, perceive your pharmacist as a medication expert, perceive your pharmacist as a health counselor, perceive your pharmacist as a patient-centered practitioner, reason for visiting a community pharmacy and Number of medications taken routinely per day.

*Reference group

VIF: Variance Inflation Factor

A second linear regression model was performed after adding the pharmacist-related variables to the previous model (as independent variables). The results showed that higher financial well-being (Beta = 0.18), a high monthly income (Beta = 7.04), perceiving the pharmacist as a medication counselor (Beta = 5.43), sometimes visiting the community pharmacy for general or specific medical advice (Beta = 3.45), visiting the community pharmacy regularly (1–5 years) (Beta = 3.06), and easy access to healthcare (Beta = 3.62) were significantly associated with a higher EQ-VAS score. A higher number of chronic diseases (Beta = −2.66), visiting the pharmacy for chronic medications (Beta = −6.20), visiting a community pharmacy weekly for medical care or counseling (Beta = −7.00), receiving counseling from the pharmacist about a medication and/or medical condition for more than 10 min (Beta = −6.31), and rarely visiting the community pharmacy to buy non-prescription medications (Beta = −3.83) were significantly associated with a lower EQ-VAS score. Moreover, in this comprehensive model, the effect size of positive pharmacist-related variables was close to 30 % of the quality of life variance, lower than that of baseline variables (55 %) (chronic diseases and socioeconomic variables) (Table 5, Model 2).

## 4 Discussion

This study included a sample of 865 adults from all Lebanese governorates and provided a proper frame of the current situation in Lebanon four years after the beginning of the crisis. Although most participants did not have chronic illnesses or problems with mobility, self-care, or usual activities, nearly half experienced some pain/discomfort and anxiety/depression, indicating that mental health and symptom burden remain pressing issues despite the relatively young mean age of our sample. The latter likely reflects the online recruitment method but also highlights that psychological distress is widespread in the Lebanese context.^30^

This study showed that overall health and well-being were better among participants with higher financial well-being (beta = 0.17), easy access to healthcare (beta = 4.16), and a high monthly income (beta = 10.92), but lower in those with chronic conditions (beta = −2.81). Oppositely, regular contacts with the community pharmacist (beta = 3.06 to 3.45) would counteract the detrimental effects of diseases on quality of life. However, given the cross-sectional design, these associations should be interpreted as correlations rather than causal effects.

We found that higher financial well-being was strongly associated with a better quality of life, consistent with studies from the United States, Europe, and Asia showing that low financial well-being and high financial stress predict poor overall health and health outcomes, and greater risks of depression and anxiety.^31^, ^32^, 33., 34. Perceived financial hardship often exerts stronger effects on self-reported health than objective measures of debt or income levels, possibly because it captures the psychosocial burden of insecurity.^31^^,^^35^ Similarly, a Lebanese study using the EQ-5D-5L scale reported that higher scores were associated with high socioeconomic status.^17^ In the same line, the current study also showed that participants with limited access to healthcare reported lower overall health and well-being. A possible explanation could be that self-reported financial well-being is related to the ability to afford healthcare costs, utilization, and preventive care, also correlated to monthly income,^36^ while participants with a low income and those who lack health coverage might be unable to afford substantial medical expenses out-of-pocket, thus leading to high levels of stress.^37^

We note that the validity of the IFDFW scale in the current Lebanese economic crisis deserves further examination, as traditional indicators of financial well-being may not fully capture the multidimensional hardship faced by households during prolonged currency collapse and health system instability.^38^^,^^39^ Comparable concerns about the scale's sensitivity have been raised during other economic downturns. For instance, evidence from the Great Recession in the UK showed that relying on average measures of life satisfaction masked significant negative impacts on vulnerable subgroups, particularly individuals who lost jobs or had preexisting health conditions.^40^

In all cases, socioeconomic difficulties should be focused on and prioritized in a country like Lebanon, which has been grappling with economic hardship, drug/device shortages, and political unrest, thus compromising the quality of care and access to care.^10^^,^^41^ Addressing financial disparities in such a context is essential to ensure the provision of equitable healthcare and promote healthy lives for all,^32^ as emphasized by the United Nations Sustainable Development Goal 3 (Good Health and Well-Being)^42^ and the International Pharmaceutical Federation (FIP) Development Goal 10 (Equity & Equality).^43^

Based on the current results, the pharmacist's role seems to be critical in enhancing well-being. Indeed, participants who perceived pharmacists as medication counselors and experts (beta = 5.43) and those who visited community pharmacies regularly (beta = 3.06) were more likely to have a better quality of life, despite an expected correlation between more visits to the pharmacy and a higher number or severity of diseases (beta = −6.20). Notably, nearly all participants living with chronic diseases regarded pharmacists as proficient in medications (94 %) and valued them as health advisors (81 %). However, counterbalancing the beneficial association of regular pharmacist counseling with better quality of life, obtaining chronic medications from the pharmacy, weekly visits to a community pharmacy for medical care or advice (beta = −7), and receiving more than 10 min of pharmacist counseling about medication (beta = −6.31) and/or medical conditions (beta = −2.66) are still associated with poorer health and well-being.

The pharmacist's role is particularly crucial in patients with numerous chronic illnesses. Participants with a higher number of chronic diseases had lower overall health and well-being, consistent with a study from Lebanon showing that diabetes mellitus, cardiovascular diseases, and an increased number of comorbidities tended to be associated with a lower quality of life.^17^ This concept was confirmed in the multivariable analyses of the current study, which showed that the effect size of positive pharmacy-related variables (standardized betas), emphasizing easy access to the pharmacist and their counseling role, remained smaller (30 %) than the association of chronic diseases and socioeconomic status with participants' quality of life (50 %): pharmacists should interpret this finding as a renewed call to duty toward the most vulnerable populations.

The central role of pharmacists identified in this study aligns with international literature demonstrating that pharmacist counseling enhances medication adherence, supports self-management, and improves chronic disease outcomes.^44^, ^45^, 46., 47, 48 Similarly, several studies from Lebanon have demonstrated the prominent role of community pharmacists as counselors in chronic diseases such as hypertension and cardiovascular diseases,^49^^,^^50^ diabetes,^50^^,^^51^ pulmonary diseases,^52^ epilepsy, and others. Indeed, pharmacists provide education and guidance to patients by supporting informed self-care related to nutrition, physical activity, hygiene, lifestyle choices, and other essential lifestyle areas. They also promote preventative measures and drive patient empowerment to make better health choices. This approach could result in better health outcomes and contribute to the sustainability of health systems and broader societal and economic gains.^49^, ^50^, 51., 52

Hence, patients with chronic diseases regularly visit community pharmacies, seeking opportunities to optimize their health outcomes. Pharmacists, for their part, can expand their counseling activities through medication therapy management (MTM) programs^53^^,^^54^ that involve a comprehensive review of patient medications, including prescription and over-the-counter drugs, to identify and resolve medication-related problems.^53^^,^^54^ Pharmacists would also screen for red flags that require a referral to a specialized medical team.^55^^,^^56^ Lebanese pharmacists showed adequate knowledge and a favorable attitude toward the implementation of medication therapy management services.^57^ However, they face several barriers, including a lack of training and inadequate staff, time, and space.^57^ Interestingly, the validation study of the Lebanese specialized competencies framework for community pharmacists (SCF-CP)^58^ showed that competency within the domain of safe and rational use of medicines had the lowest mean compared to the other competencies. Therefore, our study highlights the importance of implementing continuing professional development programs and training, especially for early career pharmacists, to equip them with the right tools to perform better in the healthcare system.^59^^,^^60^

### 4.1 Limitations and strengths of the study

Although this study has many strengths, including the use of validated scales and an adequate number of participants from across Lebanon, the possibility of bias cannot be ruled out, calling for a cautious interpretation of the results. First, the use of an online survey on a snowball convenience sample may introduce selection bias, with an overrepresentation of younger, digitally connected adults. Second, social desirability bias may have inflated positive perceptions of pharmacists, especially in a context where they are among the most accessible healthcare providers. Third, self-reported measures are prone to recall and information reporting bias. Fourth, the cross-sectional study design does not allow for causal inference due to temporal issues, and associations should be interpreted as correlations only, suggesting the need for prospective studies for better evidence. Finally, findings may not be generalizable to all Lebanese adults, particularly older or rural populations with different healthcare access patterns. And even though a multivariable analysis was performed to reduce confounding, residual confounding may still be possible. Further studies addressing these pitfalls are suggested to confirm our findings.

### 4.2 Public health implications

Our findings highlight the need for a multifaceted approach that addresses financial inequities, ensures equitable access to care, and strengthens the pharmacist's role in chronic disease management. Policy efforts should focus on improving financial protection and healthcare financing mechanisms, while simultaneously investing in pharmacist training and infrastructure for medication therapy management and preventive services, as previously suggested in the national pharmaceutical strategy.^10^ Finally, future initiatives should focus on enhancing patient education and guidance, with a strong emphasis on preventive measures and lifestyle improvement. These efforts can lead to better health outcomes, contributing to the sustainability of healthcare systems and broader societal and economic gains.

## 5 Conclusion

This study demonstrated that financial well-being, access to care, and the advisory role of pharmacists are closely linked to quality of life in Lebanon. While chronic disease burden and socioeconomic hardship remain dominant determinants of poor outcomes, pharmacists contribute positively by providing counseling and accessible care, especially in a country facing overlapping economic, social, and health crises. Strengthening pharmacists' roles through medication therapy management and continuing professional development, alongside systemic reforms to improve financial protection and healthcare access, is essential to enhance population health and well-being in crisis-affected contexts.

## CRediT authorship contribution statement

**Aline Hajj:** Writing – original draft, Investigation. **Marwan Akel:** Writing – original draft, Methodology, Investigation. **Rony M. Zeenny:** Writing – review & editing. **Hala Sacre:** Writing – review & editing, Validation. **Chadia Haddad:** Writing – review & editing, Formal analysis, Data curation. **Jihan Safwan:** Writing – review & editing. **Fouad Sakr:** Writing – review & editing. **Pascale Salameh:** Writing – review & editing, Validation, Supervision, Conceptualization.

## Consent to participate

Informed written consent was obtained from all individual participants included in the study.

## Ethics approval

This study was performed in line with the principles of the Declaration of Helsinki. The Ethics and Research Committee at the Lebanese International University approved the project (Approval number: 2023RC-013-LIUSOP).

## Ethical considerations

This study followed the ethical principles delineated in the Helsinki Declaration and received ethical approval from the Ethics and Research Committee at the Lebanese International University School of Pharmacy (2023RC-013-LIUSOP).

## Funding

The authors declare that no funds, grants, or other support were received during the preparation of this manuscript.

## Declaration of competing interest

The authors have no relevant financial or non-financial interests to disclose.

## Data Availability

The datasets generated and/or analyzed during the current study are available from the corresponding author upon reasonable request.
